# Optimization on drying conditions of a solar electrohydrodynamic drying system based on desirability concept

**DOI:** 10.1002/fsn3.168

**Published:** 2014-09-19

**Authors:** Mohammad Jafar Dalvand, Seyed Saeid Mohtasebi, Shahin Rafiee

**Affiliations:** Department of Agricultural Machinery Engineering, Faculty of Agricultural Engineering and Technology, University of TehranKaraj, Iran

**Keywords:** Desirability, EHD drying, optimization, photovoltaic energy, response surface methodology

## Abstract

The purpose of this article was to present a new drying method for agricultural products. Electrohydrodynamic (EHD) has been applied for drying of agricultural materials due to several advantages such as energy saving, low cost equipment, low drying temperatures, and superior material quality. To evaluate this method, an EHD dryer based on solar (photovoltaic) energy was designed and fabricated. Moreover, the optimum condition for the EHD drying of kiwi fruit was studied by applying the Box–Behnken design of response surface methodology. The desirability function was applied for optimization in case of single objective and multiobjective functions. By using the multiobjective optimization method, maximum desirability value of 0.865 was obtained based on the following: applied voltage of 15 kV, field strength of 5.2 kV cm^−1^, without forced air stream, and finally a combination of 17 discharge electrodes (needles). The results indicated that increasing the applied voltage from 6 to 15 kV, moisture ratio (MR) decreased, though energy efficiency and energy consumption were increasing. On the other hand, field strength of 5.2 kV cm^−1^ was the optimal point in terms of MR.

## Introduction

Conventional food drying usually uses convective, radiative, conductive, and dielectric heat transfer methods. In these methods, heat was applied to remove water from the food surface in the drying process (Alemrajabi et al. 2012). The applications of electrical treatments have shown a sustained progression in the food industries and agriculture in recent decades. Due to these difficulties, nonthermal drying methods are required. Thus, it seems necessary to determine the effectiveness of electrohydrodynamic (EHD) on time and drying rate. Based on the previous literature, EHD drying was categorized into the nonthermal drying technique (Rouaud and Havet 2009; Bai and Sun 2011). Since corona discharge can be produced at a room temperature and atmospheric pressure, this technique is particularly attractive for low-temperature applications. At present, EHD drying technology is investigated by many researchers in vegetables, fruits, and heat-sensitive materials (Bajgai and Hashinaga 2001; Cao et al. 2004; Li et al. 2006). Application of EHD drying may be a good approach to overcome the existing problems in conventional drying methods. For example, it offers low energy consumption, low cost, and high quality for food compared with hot air (convective) drying systems (Lai and Sharma 2005; Goodenough et al. 2007; Bai et al. 2011).

Response surface methodology (RSM) is a statistical procedure used for the optimization of multivariate problems (Myers et al. 2009). The optimal terms for a single response (single objective) problem are obtained easily by using surface response methodology. However, in more condition, researchers may be exposed to optimising several responses (multi objective). (Neto et al. 2006; Sivertsen et al. 2007). If optimal terms are localized in different regions with distance from each other for each response, finding the conditions in which all responses are satisfied at the same time is difficult. Therefore, changing a factor can lead to improving one specific response, simultaneously having a very negative effect on another factor. Multicriteria methodology can solve such problems. This methodology is based on creating a desirability function for every response and Derringer function is the most important in this analysis method (Murphy et al. 2005).

In this work, kiwi fruit was dried using a solar EHD drying system. The objective of this work was to study the optimum condition in the moisture ratio (MR), energy efficiency, and energy consumption for kiwi fruit drying process versus drying voltage, field strength, number of discharge electrodes (needle), and air velocity by applying a multiobjective function and Box–Behnken design of the RSM.

### Mathematical description of EHD drying

**Figure 1:**
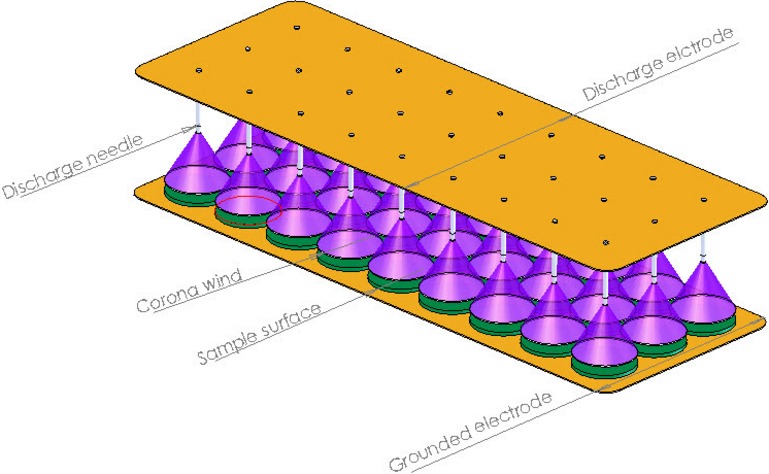
The mechanism of a corona wind.

Basically, due to the interactions of the numerous charges in a strong electric field, presenting a mathematical model for an EHD drying system is very difficult and complicated. Moreover, due to the different dielectric properties of the liquid and solid states of the material being dried, theoretical treatment is even more complex. EHD drying uses a secondary bulk flow which is known as corona wind or ionic wind. By applying a high voltage to an electrode, ionization of gas occurs due to a high electric field and thus ions are produced. These ions migrate toward the electrode plate (Fig.1) and along the electric field lines, and thus collide with air molecules, and part of their energy is used for overcoming the frictional resistance due to collisions with neutral molecules. Ion momentum is transferred from the ions to the air molecules causing air movement and thus the electric (corona) wind is produced (Fig.1).

The impingement of electric wind on wet materials produces a contact and thus improves liquid mass transfer rates from the material through increased turbulence. The velocity of the electric wind induced by a single point-to-plate EHD system can be calculated from the following equation which was derived from the conservation of energy and Gauss' laws (Cao et al. 2004):

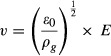
(1)
where *ε*_0_ is the electrical permittivity of workspace and is equal to 8.85 × 10^−12^ F m^−1^, *ρ*_*g*_ is the air density in kg m^−3^, and *E* is the electric field strength in kV cm^−1^. The magnitude of electric wind velocity is proportional to the electric field strength. Increase in drying rate observed due to each needle point electrodes creates electric wind (Cao et al. 2004; Alemrajabi et al. 2012).

## Materials and Methods

### Materials

Fresh kiwi fruits (cv. Hayward) were supplied from a local market. Samples were transported to the physical laboratory of the Faculty of Agricultural Engineering and Technology, University of Tehran, Karaj, Iran. Moisture contents of samples were determined based on ASAE standard (Standard 1998) and were obtained as 84% wet base prior to its use in the drying experiments. The samples were then stored in different moisture-resistant packages in a refrigerator at 2°C. The storage time was less than 3 days for all the samples. Before starting the drying process, the kiwi fruits were cut into circular slices of 3.5–5 cm in diameter and 2 mm in thickness.

### Experimental setup

The experimental setup consists of a DC high-voltage generator, grounded plate electrode, discharge point electrode (needle), heater plate, solar power supply system, and some measurement instruments. The DC high-voltage generator was designed and fabricated in the power laboratory, with an output voltage of 0–30 kV and maximum current of 2 mA with both polarities. In the present study, only positive polarity was utilized. The accuracy of the power supply was ±100 V for voltage and ±0.002 mA for current.

In the point-to-plane configuration, a vertically mounted electrode with multiple pointed needles was projected toward a fixed horizontal grounded metallic plate on which the samples to be dried were placed. The distance between the electrodes was adjustable between 0 and 8 cm. The sharp points of 17 discharge needles (0.1 mm in point diameter) were connected to a direct current high-voltage power supply that maintains a positive high voltage, vertically above the center of plane (25 × 28 cm) which was used as an electrically earthed reception plane. Electric field was applied to the samples by adjusting the voltage and also the electrode spacing. A 60 W photovoltaic module and an 18 Ah backup battery were considered for providing the required energy. In order to increase the recieived energy, the orientation of the photovoltaic module should be automatically moved toward the sun position during the day by using a designed sun tracker.

### Temperature and humidity measurements

The ranges for temperature and humidity measurements were −40 to 125°C and 0–100% relative humidity (noncondensing), respectively. The accuracy of the temperature measurement was 0.3 at 25°C. The accuracy for the humidity measurement was 2% over 10–90% relative humidity at 25°C. The resolution for the temperature measurement was 0.01°C and for the humidity measurement was 0.03% relative humidity. An infrared thermometer was used for measurement of sample surface temperature. The measurement resolution and range were 0.01°C and −70 to 380°C, respectively.

### Moisture content measurement

The slices of the samples were placed on the grounded electrode (Fig.1). To measure the weight loss of water with respect to time (i.e., the drying rate), a digital balance manufactured by A&D Corporation was used. The capacity of these types of digital balances is 0–1000 g and 0–3000 g (dual ranges) and the readabilities are 0.01 and 0.1 g, respectively.

### Methods

The initial weight and temperature of the samples were measured and then samples were prepared for drying. Ambient conditions in the laboratory during EHD drying were 24°C and 20.8% relative humidity. The samples were exposed continuously to drying during all experiments; the weight of the sample was measured at 10 min intervals. During the experimental period, the change in ambient temperature was minimal as the laboratory was well under temperature control.

The MR of kiwi fruit during drying experiments was calculated using the following equation (Toğrul and Pehlivan 2003):

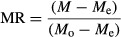
(2)
where *M*, *M*_o_, and *M*_e_ are moisture content at any drying time, initial moisture content, and equilibrium moisture content (kg water/kg dry matter), respectively. The values of *M*_e_ are relatively small compared to those of *M* or *M*_o_, hence the error involved in the simplification is negligible (Aghbashlo et al. 2008).

To evaluate the energy consumption of EHD drying process, the results are expressed in terms of consumed energy per unit of mass of moisture removed due to EHD effect as shown in the following equation:

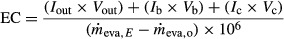
(3)
where EC is consumed energy in kJ g^−1^ 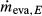 and 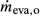 are the average rate of water evaporated due to the phenomenon EHD-enhanced and control sample in kg sec^−1^, respectively. *I*_out_ is consumed current by high-voltage electric field in ampere and *V*_out_ is applied voltage in volt. The subscripts “b” and “c” denote blower and control circuit, respectively. At the end of each experiment, the difference between the amount of water evaporated due to phenomenon EHD-enhanced and similar value for the control sample was calculated. The consumed energy per unit of mass of moisture removed was obtained by dividing the energy consumption of all components during the experiment over this value.

In order to calculate the energy efficiency of a high-voltage power supply, input and output voltage–current were measured during the experiments. Then, by applying the following equation, energy efficiency was obtained:

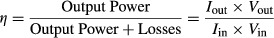
(4)
where *I* is current in ampere and *V* is applied voltage in volt. In this equation, the subscripts “in” and “out” denote input and output from the power supply, respectively.

### RSM and optimizations

Myers and Montgomery described a multiple response method called desirability. This method makes use of an objective function, D(*X*), called the desirability function. The measured properties related to each response are transformed into a dimensionless individual desirability (*d*_*i*_) scale. The desirability function reflects the desirable ranges for each response (*d*_*i*_). The scale of the individual desirability function changes between *d *=* *0, for a completely undesirable response, and *d *=* *1, for a fully desired response, above which further improvements would have no importance. This transformation makes it possible to combine the results obtained for properties measured on different orders of magnitude. The simultaneous objective function is a geometric mean of all transformed responses according to the following equation (Myers Raymond and Montgomery 2002):

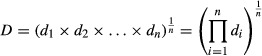
(5)
where *n* is the number of responses considered in the optimization process. Thus, the simultaneous optimization process is limited to find the levels of factors that demonstrate the maximum overall desirability. There are numerous possibilities of transformations for obtaining individual desirability. On the optimization process, the goal value for responses must be one of five choices: none, maximum, minimum, in range, or target.

The *d*_*i*_ for in range are effective in the product of the desirability function *D*, but are not considered in determining *n* by the following equation:

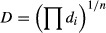
(6)

**Figure 2:**
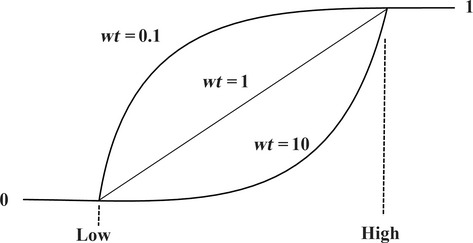
Desirability curves for goal—maximum.

However, if the goal for the response *y* is the maximum, the individual desirability (*d*) is described by the following equation (Fig.2):

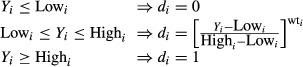
(7)

**Figure 3:**
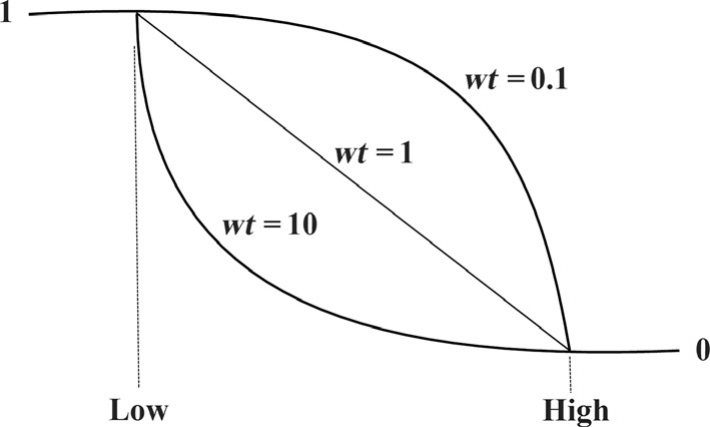
Desirability curves for goal—minimum.

If the goal is minimum, the desirability will be defined by the following equation (Fig.3):

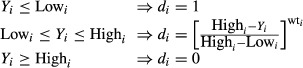
(8)
where low_*i*_ and high_*i*_ indicate the lower and higher acceptable values of the response, respectively. wt_*i*_ is the weight. Using weights emphasize the upper/lower bounds, or the target value. Thus, when wt_*i*_ = 1, the *d*_*i*_ varies from 0 to 1 in a linear form, whereas wt_*i*_ > 1 and wt_*i*_ < 1 are shown as having high and low importance in the near points of the target, respectively (Bezerra et al. 2008).

In the desirability objective function *D*(*X*), each response can be assigned an importance relative to the other responses. Importance (*r*_*i*_) varies from 1 (least important) to 5 (most important). If varying degrees of importance are assigned to the different responses, the objective function is as follows (Myers Raymond and Montgomery 2002):

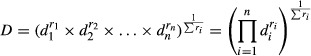
(9)
where *n* is the number of responses in the measurement. If all the importance values are the same, the simultaneous objective function reduces to the normal form for desirability.

In order to examine the combined effects of the four different factors (independent variables): air velocity, applied voltage, field strength, and number of discharge needle on the EHD drying process of kiwi fruit (MR, energy efficiency, and energy consumption) and to derive an optimum model, RSM was used. A four-factor and three-coded level (−1, 0, and +1) Box–Behnken design was used to determine the optimum condition in the EHD drying process. There were six replicates at the center points and single runs for each of the other combinations; 30 runs were done in a totally random order. In order to optimize the drying process, constraints for desirability function were defined based on energy consumption, efficiency, and MR with emphasis on energy parameters as presented in Table1.

**Table 1:** Constrains in desirability function.

Parameter	Unit	Goal	Lower limit	Upper limit	Lower weight	Upper weight	Importance
*V*	kV	In range	6	15	1	1	3
*E*	kV cm^−1^	In range	3	6	1	1	3
*N*	–	In range	1	17	1	1	3
*U*	msec^−1^	In range	0	0.4	1	1	3
MR	–	Minimize	0.315	0.657	1	1	3
Efficiency	%	Maximize	3.01	29.59	2	1	3
Energy	kJ g^−1^	Minimize	0.73	10.61	1	2	3

*V* is the applied voltage in kV, *E* is field strength in kV cm^−1^, *N* represents the number of discharge needles, and *U* is air velocity in msec^−1^.

## Results and Discussion

### Single objective optimization

**Figure 4:**
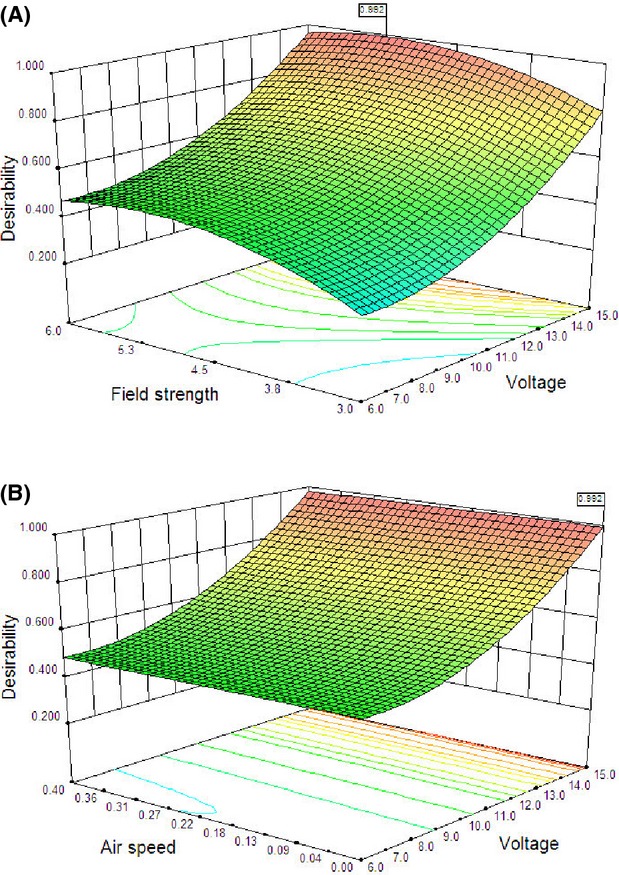
Response surface plots for the effect of (A) applied voltage and field strength; (B) applied voltage and number of discharge needles on moisture ratio desirability function.

**Figure 5:**
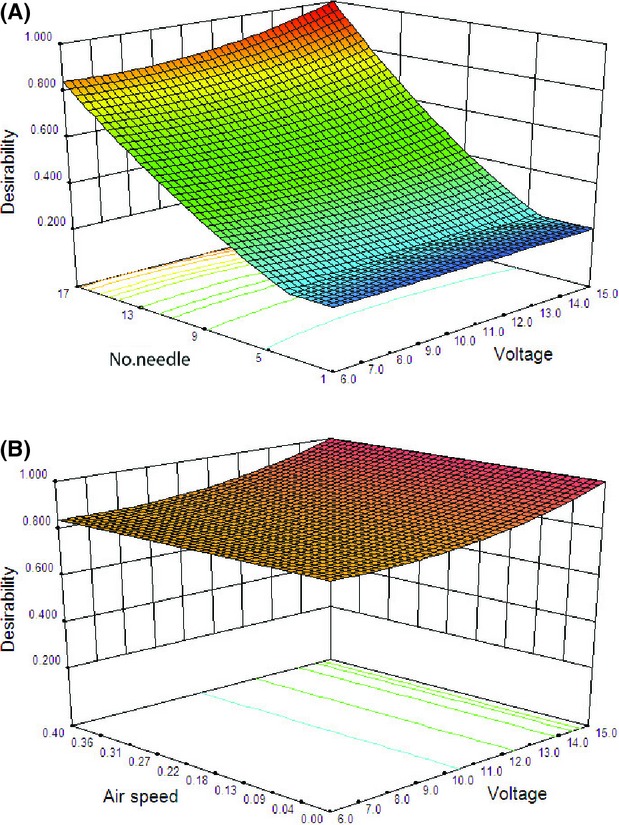
Response surface plots for investigating the effect of (A) applied voltage and number of discharge needles, and (B) applied voltage and air velocity on efficiency desirability function.

**Figure 6:**
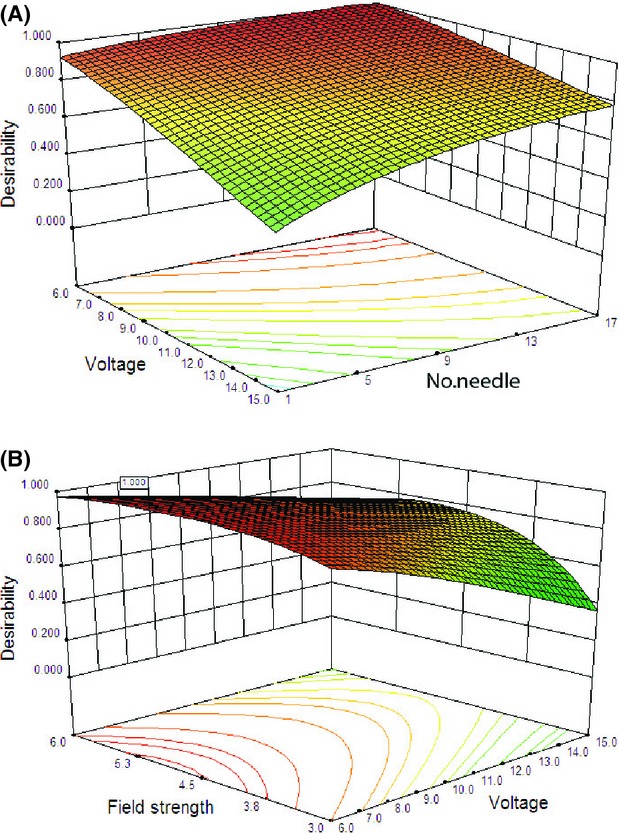
Response surface plots to investigate the effect of (A) applied voltage and number of discharge needles, and (B) applied voltage and field strength on energy consumption desirability function.

The main goal of the response surface is to find efficiently the optimum values of the variables such that the response is maximized. Each contour curve represents an infinitive number of combinations of two test variables with the other two maintained at their respective medium level. The maximum predicted value is indicated by the surface confined in the smallest ellipse in the contour diagram. Elliptical contours are obtained when there is a perfect interaction between the independent variables (Muralidhar et al. 2001). The effects of independent variables on optimizing the EHD drying process with desirability function were studied by plotting three-dimensional response surfaces against any two independent variables, while keeping another variable in its respective medium level. The three-dimensional response surfaces curves for the single response method are plotted in Figures4, 5, and 6. Responses include MR, energy consumption, and efficiency of power supply. The corresponding contour plots, represented by the projection of the response surfaces in the *x* and *y* planes, provide a straightforward determination of the effects of the independent variables on the dependent variable.

As shown in Table1, minimization was the target value of desirability function for MR. Figure4A shows the simultaneous effect of applied voltage and field strength on the desirability function of MR. The desirability function was found to be very sensitive to changes in applied voltage. The desirability was sharply increasing when the applied voltage was increased from 6 to 15 kV. On the other hand, the results showed that the maximum desirability was achieved at a field strength of 5.2 kV cm^−1^ and an applied voltage of 15 kV, whereas under these conditions, the maximum evaporation rate occurred. Bai et al. investigated the EHD drying process of Scallop. They reported that drying rate increases with increasing applied voltage. Moreover, they reported that higher voltage between the two electrodes led to a stronger electric field, and also faster drying rate. With 45 kV applied voltage, the rate of high-voltage electric field drying can be improved by 7.17 times compared with 0 kV (Bai et al. 2012). The drying rate of tomato slices treated by a high-voltage electrostatic field (HVEF) was investigated by Esehaghbeygi and Basiry. The drying rate of tomato slices was studied in three levels of applied voltages (6, 8, and 10 kV). They reported that as the voltage increases, the drying rate also increases (Esehaghbeygi and Basiry 2011). Selected points with the highest desirability values for MR response are shown in Table2. The results indicated that the condition consists of applied voltage of 15 kV, field strength of 5.2 kV cm^−1^, air velocity of 0 msec^−1^, and just one needle that had the highest desirability value, suggesting that maximum evaporation occurs in these conditions.

**Table 2:** Configuration of independent and dependent parameters in selected desirability.

Constrain	*V* (kV)	*E* (kV cm^−1^)	*U* (msec^−1^)	*N*	Energy (kJ g^−1^)	MR	Efficiency (%)	Desirability
Minimize MR	15	5.2	0	1	5.019	0.317	4.91	0.992
Maximize efficiency	15	6.0	0.4	17	3.444	0.387	29.63	1.000
Minimize energy	6.4	5.1	0.1	15	0.715	0.555	20.47	1.000

The desirability value of energy efficiency as a function of applied voltage and the number of discharge needles (Fig.5A) and applied voltage and air velocity (Fig.5B) are plotted in Figure5. The target value was to reach maximum energy efficiency. Air velocity indirectly affects efficiency, and thus it does not have an important affecting parameter on energy efficiency of power supply (Fig.5B). Moreover, increasing number of discharge needle leads to higher desirability value. There is a direct relationship between the amount of load and energy efficiency of high-voltage power supply and finally, the efficiency of the power supply reached 90% under saturation conditions. According to Table1, the energy efficiency is seen to change from 3% to 30%. Thus, it is obvious that increasing the number of discharge electrodes leads to increase in efficiency. It can be concluded that these results are exactly opposite to the results obtained for MR desirability function. The results indicating optimal values for each response are localized in different regions, and thus it is more difficult to find the conditions that simultaneously satisfy all responses.

The desirability value of energy consumption as a function of applied voltage and the number of discharge needles (Fig.6A) and applied voltage and field strength (Fig.6B) are plotted in Figure6. As seen in the figure, with an increase in applied voltage, the desirability value further decreased to 0.50 (Fig.6A). Moreover, the response curves did not show any curvature; rather, they were flattened with more and more points moving toward a higher desirability value (Fig.6A). Thus, the surface plots suggested a demand for lower applied voltage along with more needle numbers to obtain maximum desirability value. From Figure6B, it can be concluded that the curve shows the desirability that did not vary as much as the field strength in low applied voltage. In other words, with the increase in applied voltage, its effect becomes more visible. This could be due to the measurement error. Due to the energy consumption in low applied voltage, the voltage level was very low and therefore full-scale error of measurement device can happen in this condition. The selected point with the highest desirability value for energy consumption response is shown in Table2.

### Multiobjective optimization

**Figure 7:**
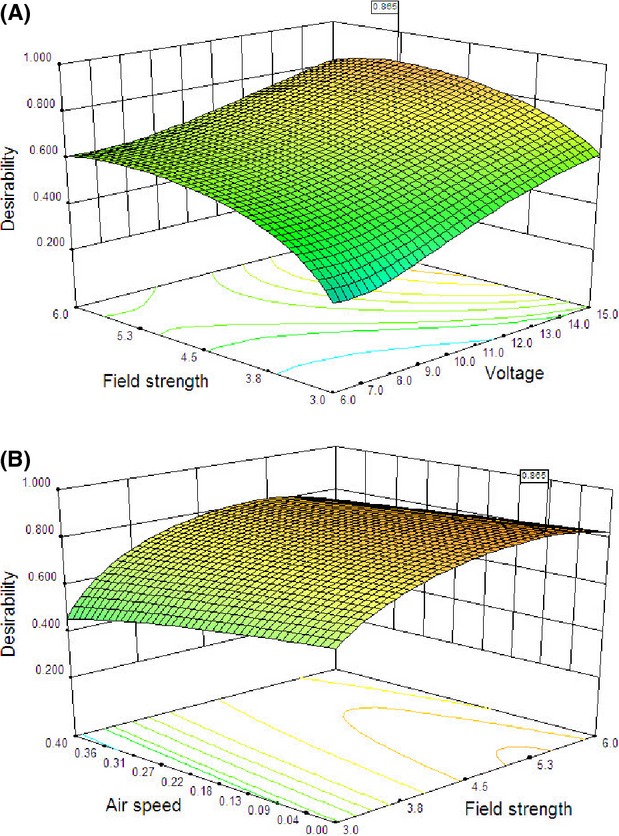
A three-dimensional response surface plot illustrating optimal conditions for reciprocal of desirability as a function of (A) applied voltage and field strength, and (B) field strength and air velocity.

**Figure 8:**
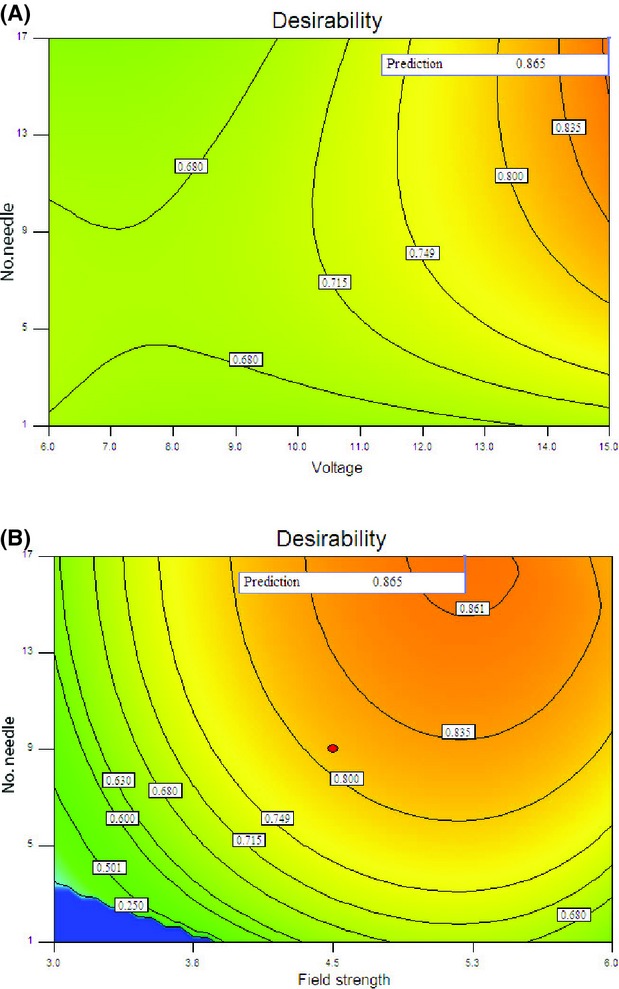
Contour plot illustrating optimal conditions for reciprocal of desirability as a function of (A) applied voltage and number of discharge needles, and (B) field strength and number of discharge needles.

MR, energy efficiency, and energy consumption were chosen as responses. Moreover, due to the influence of EHD drying on reducing energy consumption, the value of 2 for the weight of efficiency and energy consumption was considered (Table1). The three-dimensional response surface and the two-dimensional contour plots are the graphical representations of the regression equation. Both plots are presented in Figures7 and 8.

Generally, desirability increases with increasing applied voltage and number of discharge needles (Figs.7A, 8A) and decreases with increasing air velocity (Fig.7B). As presented in Figure7A, the highest value of desirability function was observed in the field strength of 5.2 kV cm^−1^, and desirability increases with increasing field strength from 3 to 5.2 and decreases with more increasing field strength up to 6 kV cm^−1^. There was an optimal point in field strength of 5.2 kV cm^−1^. It seems that the air velocity had the least effect on desirability function in comparison with other variables and also, it was expected to have an enhancer effect on the drying process; however, it did not have such an effect (Fig.7B). Perpendicular mixing of electrical and forced air flows is probably responsible for reducing the effect of forced air flow on the drying process.

Although increasing the number of discharge needles almost enhanced the desirability value, variation in desirability value due to effect of applied voltage was much more than effect of the number of discharged needle (Fig.8A).

The response surface is completely elliptical as shown in Figure8B by using the number of needle and field strength as independent variables. Maximum desirability in this case was predicted to be 0.865, corresponding to the maximum level of number of needle. The changes in the drying process of an okara cake dried with an EHD technique in an oven at 105°C were investigated by Li et al. They reported that the effect of the multiple point-to-plate system on the drying rate was not integral times higher than the single point-to-plate system. The difference in results is probably due to the differences in the electrode configuration, test temperature, and type of product. Changing the needle's position may lead to a superposition of their fields (Li et al. 2006).

The final results of desirability function for three responses optimization are given in Table3. As presented in Table3, maximum desirability (bold Item) was equal to 0.865 and the conditions to achieve it were applied voltage of 15 kV, field strength of 5.2 kV cm^−1^, air velocity of 0 msec^−1^, and a combination of 17 discharge needles. This indicates that it would be possible to achieve a higher desirability value if the applied voltage and number of discharge needles increase beyond the upper limit of their ranges. Alemrajabi et al. (2012) studied the EHD drying process of a carrot slice, and reported that the electric field strength of 5.2 kV cm^−1^ led to a maximum evaporation of carrot slice. In a similar experiment, Hashinaga et al. (1999) reported the optimum field strength to be ˜4.7 kV cm^−1^ in EHD drying of apple slices by using AC voltages.

**Table 3:** Final solutions of desirability.

Run	*V* (kV)	*E* (kV cm^−1^)	*U* (msec^−1^)	*N*	Energy (kJ g^−1^)	MR	Efficiency (%)	Desirability
1	**15**	**5.2**	**0**	**17**	**2.381**	**0.388**	**28.70**	**0.865**
2	15	5.3	0	17	2.367	0.388	27.01	0.864
3	15	5.1	0	16	2.466	0.388	27.56	0.864

The bold item showed a maximum desirability value.

**Figure 9:**
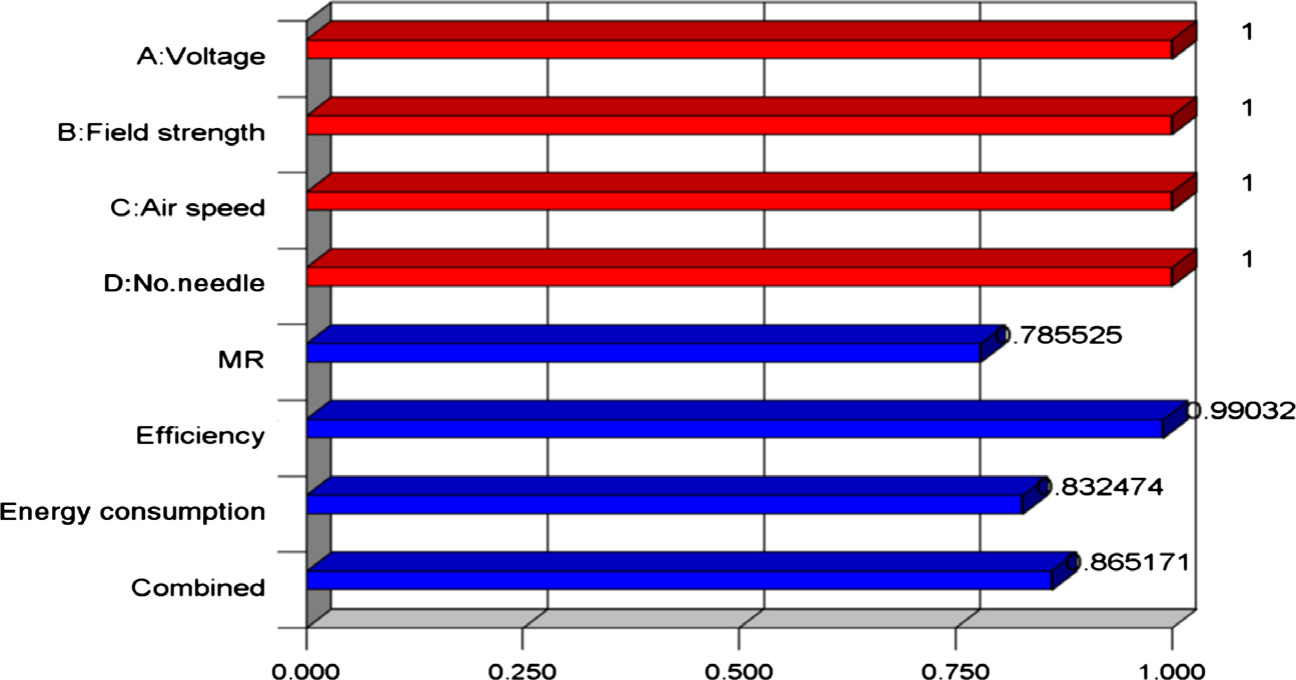
Distribution of desirability on the desirability function.

As discussed earlier, for optimizing several responses, simultaneous objective function is a geometric mean of all transformed responses, and Figure9 shows the desirability of each response. The highest and lowest desirability were observed for MR and efficiency, respectively. This means that efficiency is closer to its target value as compared to MR.

## Conclusion

The advantages of optimization based on RSM were discussed. By using the Box–Behnken design, the number of required experiments was obtained to be more than two times and less than that of the full factorial approach. Applying the Box–Behnken design decreases the time of analysis and also the experimental expense without any obvious reduction in efficiency. Moreover, optimization using RSM methodology was a useful tool for process optimization. The optimization of drying condition resulted in a reduced cost. The chosen method of optimization was efficient, relatively simple, and time- and material-saving.

Generally, the results indicated that it would be possible to achieve a higher desirability value if the applied voltage and the number of discharge needles increase beyond the upper limit of their ranges. However, increasing the number of discharge needles leads to scaling up of the system. Therefore, this could be promising for its industrialization.
